# Obesity induced by a high-fat diet regulates the MYC‒PPIL1 network in the mediation of asthenozoospermia

**DOI:** 10.1186/s12610-025-00289-8

**Published:** 2025-10-10

**Authors:** Menghua Shi, Lei Xu, WeiXi Zheng, Xuyao Lin, Guozheng Qin

**Affiliations:** 1 First Clinical Medical College, Yunnan University of Chinese Medicine, Kunming, China; 2 Yunnan Provincial Hospital of Chinese Medicine/The First Affiliated Hospital of Yunnan University of Chinese Medicine, Kunming, 650021 China

**Keywords:** High-fat diet, Asthenozoospermia, MYC, PPIL1, L-carnitine, Régime riche en Graisses, Asthénozoospermie, MYC, PPIL1, L-carnitine

## Abstract

Male infertility, particularly asthenozoospermia (AZS), has become an increasingly severe global public health issue. Obesity induced by a high-fat diet (HFD) is considered a key factor in the development of AZS, although its exact molecular mechanisms remain unclear. This study employs bioinformatics analysis to explore the key genes and potential regulatory mechanisms of HFD-induced obesity in AZS, which were validated by animal experiments. First, on the basis of GEO transcriptomic data, we identified nine common differentially expressed genes (DEGs) between HFD-induced obesity and AZS. Using LASSO regression and support vector machine methods, we subsequently identified C1QBP and PPIL1 as critical genes associated with HFD-induced AZS. Furthermore, a core gene‒transcription factor coexpression network revealed that MYC is likely an upstream transcriptional regulator of these two core genes. According to single-cell RNA-seq data, C1QBP and PPIL1 are predominantly expressed in spermatogonia, whereas MYC is primarily localized in stromal cells and is closely correlated with AZS. Additionally, through genome-wide enrichment analysis, we identified several key pathways regulating both HFD-induced obesity and AZS, including cell proliferation and differentiation (MYC targets and mTOR signalling), energy metabolism, cellular stress and homeostasis, and immune and inflammatory responses. The results of animal experiments demonstrated that HFD-induced obesity significantly impaired sperm motility in male rats, accompanied by decreased testosterone levels and increased oxidative stress. At the molecular level, the expression of MYC and mTOR in the HFD obesity/AZS group was significantly reduced (*P* < 0.01), whereas PPIL1 expression was significantly increased (*P* < 0.01). Moreover, L-carnitine partially reversed these changes, indicating potential therapeutic value. In conclusion, our study suggests that HFD-induced obesity may lead to AZS through the upregulation of PPIL1 levels and the inhibition of the MYC and mTOR signalling pathways.

## Résumé

**Contexte:**

L’infertilité masculine, en particulier l’asthénozoospermie (AZS), est devenue un problème de santé publique mondial de plus en plus grave. L’obésité induite par un régime riche en graisses (HFD) est considérée comme un facteur clé dans le développement de l’AZS, bien que les mécanismes moléculaires exacts restent incertains. Cette étude utilise l’analyse bio-informatique pour explorer les gènes clés et les mécanismes de régulation potentiels de l’obésité induite par HFD dans l’AZS, qui ont été validés par des expériences sur des animaux.

**Résultats:**

Tout d’abord, sur la base des données transcriptomiques GEO, nous avons identifié neuf gènes différentiellement exprimés (DEG) communs entre l’obésité induite par HFD et l’AZS. À l’aide de méthodes de régression LASSO et de machine à vecteurs de support, nous avons par la suite identifié C1QBP et PPIL1 comme des gènes critiques associés à l’AZS induit par HFD. De plus, un réseau de co-expression gène-facteur de transcription a révélé que MYC est probablement un régulateur transcriptionnel en amont de ces deux gènes principaux. Selon les données de séquençage de l’ARN sur cellule unique, C1QBP et PPIL1 sont principalement exprimés dans les spermatogonies, tandis que MYC est principalement localisé dans les cellules stromales et est étroitement corrélé à l’AZS. De plus, grâce à l’analyse de l’enrichissement à l’échelle du génome, nous avons identifié plusieurs voies clés régulant à la fois l’obésité induite par HFD et l’AZS, y compris la prolifération et la différenciation cellulaires (cibles MYC et signalisation mTOR), le métabolisme énergétique, le stress cellulaire et l’homéostasie, et les réponses immunitaires et inflammatoires. Les résultats des expériences animales ont montré que l’obésité induite par HFD altérait considérablement la motilité des spermatozoïdes, accompagnée d’une diminution des niveaux de testostérone et d’une augmentation du stress oxydatif. Au niveau moléculaire, l’expression de MYC et de mTOR dans le groupe obésité HFD/AZS a été significativement réduite (p<0,01), alors que celle de PPIL1 a été significativement augmentée (p<0,01). Enfin, la L-carnitine a partiellement inversé ces changements, indiquant une valeur thérapeutique potentielle.

**Conclusions:**

Notre étude suggère que l’obésité induite par HFD peut conduire à l’AZS par la régulation positive des niveaux de PPIL1 et l’inhibition des voies de signalisation MYC et mTOR.

## Introduction

Male infertility is a significant global health issue. According to the World Health Organization (WHO), approximately 10.1% to 15.8% of couples of reproductive age face infertility, with male factors contributing to approximately 50% of cases [1]. Among these, asthenozoospermia (AZS)—characterized by a sperm motility rate of less than 32% with otherwise normal semen parameters—represents a predominant condition, accounting for about 20% to 40% of male infertility cases [2, 3]. The etiology of AZS is multifactorial, involving genetics, environmental pollutants, occupational exposures, and lifestyle factors such as dietary habits and obesity [4, 5]. In particular, obesity has emerged as a major public health concern and a key risk factor for the decline in male fertility. It not only predisposes individuals to various metabolic diseases but also contributes to hormonal imbalances, impaired spermatogenesis, reduced semen quality, and sexual dysfunction, thereby significantly increasing the risk of male infertility [5, 6]. Critically, the detrimental effects of high-fat diet (HFD)-induced obesity on sperm motility and overall fertility are increasingly recognized and cannot be overlooked.

Obesity induced by a high-fat diet (HFD) has emerged as a critical risk factor for AZS, disrupting endocrine homeostasis, promoting oxidative stress, and impairing sperm mitochondrial function. Epidemiological evidence consistently demonstrates that obese men exhibit poorer semen quality, including reduced sperm concentration and motility, alongside lower testosterone levels [7, 8].At the molecular level, HFD-induced obesity dysregulates key signaling pathways involved in spermatogenesis and energy metabolism, such as mTOR and MYC, which are crucial for sperm production and function [9–12]**.** Additionally, HFD exacerbates testicular oxidative stress and inflammation, further compromising sperm motility and DNA integrity [5]**.**

Although the association between HFD-induced obesity and AZS is well-established, the precise molecular networks and transcriptional regulators underlying this relationship remain incompletely elucidated. Recent advances in transcriptomics and bioinformatics offer powerful tools to identify shared gene signatures and pathways linking obesity to male infertility. In particular, the role of MYC as a transcriptional regulator in both adipogenesis and spermatogenesis, as well as its potential interaction with spliceosome components such as PPIL1, warrants further investigation.

Furthermore, interventions targeting oxidative stress and metabolic dysregulation represent promising therapeutic strategies. L-carnitine, a natural antioxidant and essential cofactor in fatty acid metabolism, has shown efficacy in improving sperm motility and testicular function in both clinical and preclinical studies [13–15]^.^ For instance, recent research demonstrated that L-carnitine supplementation ameliorates HFD-induced sperm defects by enhancing mitochondrial function and reducing oxidative damage [16]. These findings provide a strong rationale for investigating the therapeutic potential of L-carnitine in the context of HFD-induced AZS.

In this study, we integrated bioinformatics analyses with experimental validation to identify key genes and pathways through which HFD-induced obesity contributes to AZS. We hypothesized that the MYC–PPIL1 axis plays a central role in this process and that L-carnitine may exert protective effects by modulating this network. Our findings aim to provide novel insights into the molecular mechanisms of obesity-related male infertility and identify potential targets for intervention.

## Methods

### Data acquisition

Transcriptomic datasets related to asthenozoospermia (AZS) and high-fat diet (HFD)-induced obesity were retrieved from the GEO database. The GSE160749 and GSE34514 datasets included RNA transcriptomic data from 9 AZS patients and 10 normal controls. The GSE28005 dataset comprises data from 5 HFD-induced obese patients and 5 normal controls. The GSE26982 and GSE8700 datasets were used as external validation sets for AZS and HFD, respectively.

### Differential gene screening

The limma package in R software was used to normalize and log2 standardize the data from independent studies to identify differentially expressed genes (DEGs) [17]. The threshold for selecting DEGs in AZS was set at |log2FC|> 0.37, *p* < 0.05, whereas for HFD-induced obesity, the threshold was |log2FC|> 1.0, *p* < 0.05. The DEGs of AZS and HFD subsequently overlapped to identify potential common targets for HFD-induced obesity and AZS comorbidity.

### Identification and validation of core genes

In the process of feature selection and model training for core genes, we applied two machine learning methods: LASSO regression and support vector machine (SVM) methods. First, we used the glmnet package in R for LASSO regression to eliminate redundant factors, employing threefold cross-validation for gene selection. SVM analysis was then performed via the e1071 and caret packages in R to identify the genes that contributed the most to group differences. Next, by overlapping the core genes identified by the two methods, we identified potential diagnostic biomarkers common to both HFD-induced obesity and AZS. To validate the diagnostic ability of the core genes, we generated receiver operating characteristic (ROC) curves on both internal and external datasets.

### Core gene–transcription factor coexpression network construction

To investigate the regulatory mechanisms of the core genes, we used the igraph package in R to construct a core gene–transcription factor coexpression network, predicting key upstream transcription factors that interact with the core genes. The core gene–transcription factor coexpression network was then visualized via Cytoscape (v3.9.1). Additionally, we used the Male Health Atlas (MHA) database to analyse the expression and localization features of the core gene–transcription factor network in testicular tissue cell populations.

### GSEA analysis

Gene set enrichment analysis (GSEA) was performed via GSEA 3.0 software. We downloaded the h.all.v7.4.symbols.gmt subset from the Molecular Signatures Database to assess related pathways and molecular mechanisms. On the basis of the gene expression profiles and phenotypic groupings from HFD-induced obesity and AZS genome-wide data, we set the minimum gene set to 5, the maximum gene set to 5000, and 1000 resamplings.

### Experimental materials

Thirty-six male Sprague–Dawley (SD) rats (170 ± 10 g) were purchased from Beijing SiPeiFu Laboratory Animal Co., Ltd. (Licence No. SCXK (Jing) 2019–0010). The rats were housed at the Animal Experiment Center of Yunnan University of Chinese Medicine (SYXK(Dian)2022–0004). All experiments were conducted in accordance with the ethical guidelines of the Animal Ethics Committee of Yunnan University of Chinese Medicine (Ethical Approval No. R-062023204).

Standard chow was purchased from Beijing SiPeiFu Laboratory Animal Co., Ltd., and a high-fat diet (HFD) was purchased from Yunnan Zhili Technology Co., Ltd. (formula: basal feed + 18% lard + 18% fish meal + 15% sugar + 2% cholesterol + 0.2% bile salts). L-carnitine (Northeast Pharmaceutical Shenyang First Pharmaceutical Co., Ltd.; batch no. Guo Yao Zhun Zi H19990372; product name: Dongwei Li; specification: 10 ml:1 g) was used.

### Modelling and drug intervention

The 36 male SD rats were randomly divided into two groups: a normal diet group (control group) with 12 rats and a high-fat diet group (model group) with 24 rats, which were fed a high-fat diet for 12 weeks. The water and food were changed daily, and the body weights of the rats were measured weekly.

The criterion for successful obesity induction by a HFD was that the body weight increased by more than 20% compared with that of the control group [18, 19]. The criteria for the successful establishment of the HFD-induced AZS model were as follows: sperm motility in the HFD obese group was significantly lower than that in the control group. L-carnitine (LC) has been shown to improve obesity and sperm motility [13–15]. After the model was successfully established, LC was administered by gavage at a dosage of 0.18 g/kg·d, which was based on the ratio of rat to human body surface area, for 8 weeks [20].

### Sample collection

Blood samples from rats were collected from the abdominal aorta. The anaesthetized rats were placed supine on a board, with their incisors and limbs fixed. After routine disinfection of the abdomen, surgical scissors were used to open the abdominal midline, exposing the abdominal cavity. Using fine forceps, surrounding tissues were separated from the blood vessels, and excess fat covering the blood vessels was wiped away with cotton. Once the abdominal aorta was fully exposed, a needle was inserted towards the heart at a depth of approximately 5 mm, and blood was collected in a vacuum tube, with 8–10 ml of blood collected from each rat. The blood was left to stand for more than 4 h and then centrifuged for 15 min at 4000 rpm. The supernatant was transferred to a 1.5 ml EP tube and stored at −80 °C for subsequent ELISA analysis.

The scrotum was carefully opened along the midline, revealing oval-shaped testes. The surrounding tissues were separated via blunt dissection, and the testes and epididymides were removed. Excess tissue was washed in a beaker containing sterile physiological saline to remove blood, and the surrounding fatty tissue was carefully trimmed. The weights of the testes and epididymides were recorded. Sperm were collected from the epididymides for sperm quality analysis. One testis was fixed in 4% paraformaldehyde for subsequent pathological examination, while the other testis was stored in liquid nitrogen for later molecular biology experiments.

### Measurement of Indicators

#### Organ Index

The testes and epididymides were collected, excess surface moisture was blotted off, and the organ weights were recorded. The organ index was calculated as follows: Testes/Epididymides = (Average weight of both testes/Average weight of both epididymides)/Body weight × 100%. The left testis was fixed in neutral formalin, and the right testis was stored at −80 °C for later use.

#### Sperm motility detection

After fat was removed from the epididymides, the epididymides were washed with saline several times. One side of the epididymis tail was excised and placed in a 5 ml EP tube. Three milliliters of saline were added to the EP tube, and the epididymal tail was cut and placed in a 37 °C water bath for 30 min. After incubation, 10 μL of the solution was dropped onto a counting chamber, and sperm motility was assessed under a 400 × light microscope by counting sperm motility in the four corners of the chamber. The total sperm motility per milliliter of sperm suspension was calculated according to methods in the literature [21].

#### Hematoxylin and eosin (H&E) staining

Testicular tissue was fixed in 4% paraformaldehyde for 24 h, dehydrated, cleared, infiltrated with paraffin, and embedded in paraffin blocks. The paraffin sections were subjected to routine dewaxing and hydration. After being washed with distilled water, the tissue was stained with hematoxylin and eosin at room temperature. The sections were dehydrated via graded ethanol, followed by 5 min of dehydration in anhydrous ethanol and 5 min of clearing in xylene. The sections were mounted with neutral resin and examined under a standard optical microscope for imaging.

#### Spermatogenic cell apoptosis (TUNEL) assay

Testicular tissue was prepared as paraffin sections, and the sections were incubated with 20 μg/ml proteinase K (without DNase) at room temperature for 30 min. After washing, the sections were incubated with 50 μL of TUNEL detection solution at 37 °C in the dark for 60 min, followed by incubation with DAPI staining solution at room temperature for 5 min. After being washed with PBS, the sections were mounted with anti-fade mounting solution, and the results were observed under a fluorescence microscope.

#### ELISA

Serum levels of hormones (T, E2) and oxidative stress markers (SOD, CAT) were measured following the manufacturer’s instructions for the ELISA kits.

#### Testicular ROS detection

Frozen sections were treated with ROS staining solution and incubated in a dark incubator at 37 °C for 30 min. After being washed with PBS three times (3–5 min each), the slides were air-dried and incubated with DAPI staining solution in a dark incubator at 37 °C for 10 min. The slides were then washed three times in PBS (3–5 min each), air-dried, and mounted with antifade mounting medium. The slides were immediately observed under a fluorescence microscope to assess the red fluorescence of ROS in testicular tissue, and images were captured for analysis.

#### Western Blotting (WB)

Approximately 50 mg of testicular tissue was homogenized with RIPA lysis buffer, phosphatase inhibitor, PMSF, and cocktail on ice. After centrifugation at 12,000 rpm for 20 min, the supernatant was collected, and the protein concentration was determined via the BCA protein assay. Equal amounts of protein were mixed with 5 × loading buffer and boiled for 10 min for protein denaturation. The proteins were separated by SDS‒PAGE, transferred onto a membrane, and washed with TBST. The membranes were blocked with 5% nonfat milk for 1 h at room temperature. The membrane was incubated with primary antibodies against PPIL1 (18 kDa, rabbit polyclonal antibody, 1:1000; Proteintech; RRID: AB_2169603; Cat# 15,144–1-AP), C1QBP (32 kDa, rabbit polyclonal antibody, 1:1000; Proteintech; RRID: AB_2827427; Cat# 24,474–1-AP), MYC (50 kDa, rabbit polyclonal antibody, 1:2000; Proteintech; RRID: AB_2148585; Cat# 10,828–1-AP), mTOR (289 kDa, rabbit polyclonal antibody, 1:1000; Proteintech; RRID: AB_2881102; Cat# 28,273–1-AP), and GAPDH (36 kDa, rabbit polyclonal antibody, 1:500; Servicebio; RRID: AB_2943040; Cat# GB15004-100) at 4 °C overnight. After incubation, the membrane was washed with TBST and incubated with the secondary antibody IRDye® 680RD goat anti-rabbit IgG (1:5000; LI-COR; RRID: AB_2721181; Cat# P/N 925–68071P) for 1 h in the dark at room temperature. The membrane was washed again, and the fluorescence intensity was detected via an infrared fluorescence imaging system. The protein bands were analysed via ImageJ to calculate the ratio of target protein expression to that of the internal reference protein GAPDH.

#### Statistical analysis

All statistical analyses were performed using SPSS 27.0 software. Normally distributed measurement data are presented as mean ± standard deviation (x̄ ± s). One-way analysis of variance (One-way ANOVA) was used for comparisons among multiple groups. If homogeneity of variance was met, the Least Significant Difference (LSD) test was applied for post-hoc pairwise comparisons; otherwise, Tamhane's T2 test was used. Comparisons between two groups were conducted using independent samples t-test. A P-value < 0.05 was considered statistically significant.

To further investigate the underlying molecular mechanisms, this study incorporated bioinformatics analysis methods. Relevant transcriptome datasets were obtained from the GEO database, and the limma package in R language was used to identify differentially expressed genes (DEGs). Least Absolute Shrinkage and Selection Operator (LASSO) regression algorithm and Support Vector Machine (SVM) model were further employed for feature selection of common DEGs to identify core genes. Gene Set Enrichment Analysis (GSEA) was performed to explore significantly enriched biological pathways. Additionally, Cytoscape software was applied to construct a transcription factor-core gene regulatory network to predict upstream regulatory relationships. Statistical graphs were generated using GraphPad Prism 8.0 software.

## Results

### Differential gene screening

**Fig. 1:**
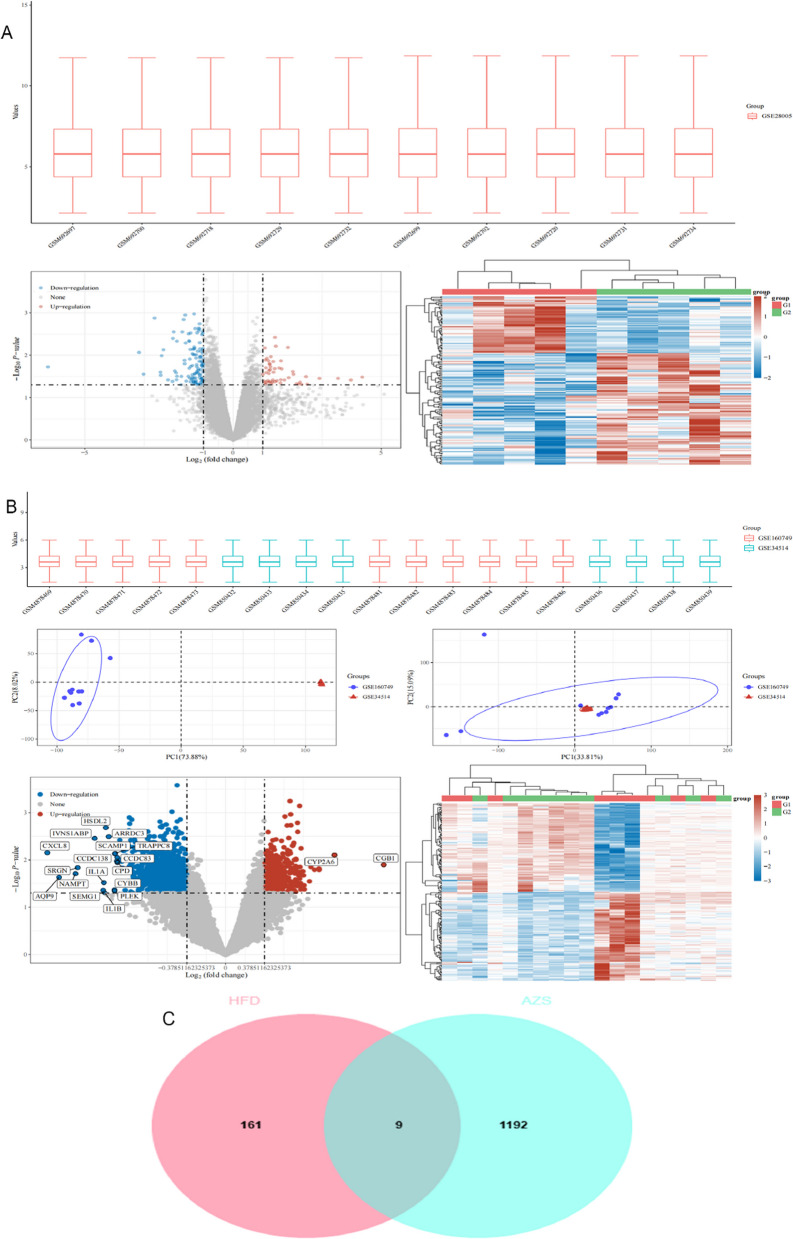
Identification of differentially expressed genes (DEGs) associated with high-fat diet (HFD) and asthenozoospermia (AZS). **A** DEGs induced by high-fat diet (HFD) obesity. **B** DEGs in asthenozoospermia (AZS). **C** Venn diagram of genes shared between HFD-induced obesity and AZS. The x-axis represents log₂ fold change, and the y-axis shows the -log₁₀ P-value. Points in red indicate up-regulated genes, blue indicates down-regulated genes, and grey represents non-significant genes

The GSE160749, GSE34514 and GSE28005 datasets were downloaded from the GEO database to screen for differentially expressed genes (DEGs) in AZS and HFD-obese patients. The "limma" package was used to normalize and standardize the datasets, resulting in 170 DEGs related to HFD obesity, including 51 upregulated genes and 119 downregulated genes (Fig. 1A). For AZS, 1201 DEGs were identified, with 426 upregulated genes and 775 downregulated genes (Fig. 1B). The number "161" in the "HFD" circle represents the number of DEGs unique to HFD.The number "1192" in the "AZS" circle represents the number of DEGs unique to AZS.The overlapping DEGs between AZS patients and HFD-obese patients revealed 9 key genes (AACS, LOXL1, COL5A3, ZGPAT, C1QBP, PPIL1, MCRIP2, NAMPT, and SERPINA3) (Fig. 1C).

### Identification and validation of core genes

**Fig. 2:**
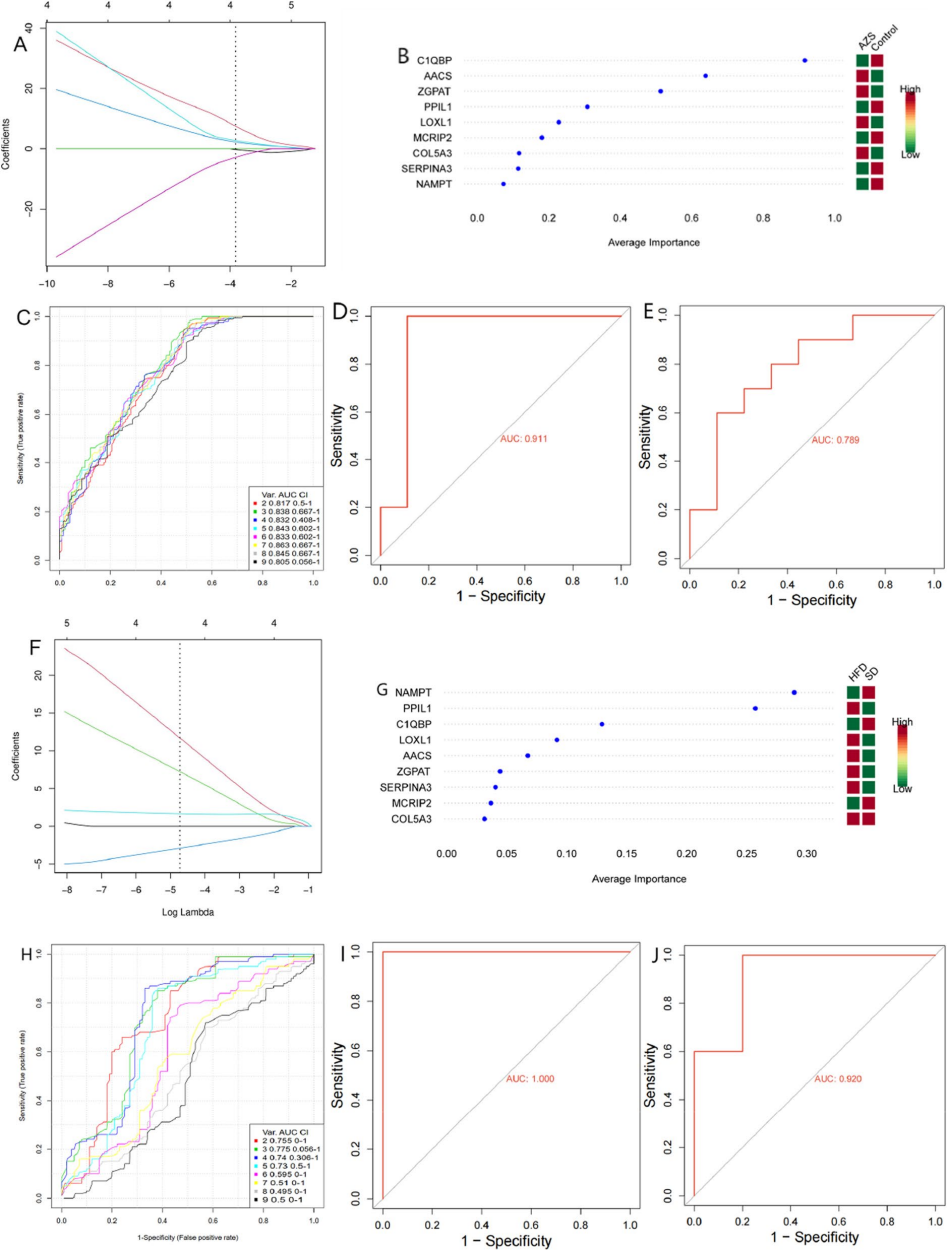
Hub Gene Screening and Validation (**A**) LASSO regression model algorithm for AZS. **B**-**C** Support vector machine (SVM) algorithm for AZS. **D**-**E** ROC validation curves for the internal/external datasets of AZS. **F** LASSO regression model algorithm for HFD obesity. **G**-**H** Support vector machine (SVM) algorithm for HFD obesity. **I**-**J** ROC validation curves for internal/external datasets of HFD obesity

First, we used LASSO regression and support vector machine (SVM) methods to identify 9 key genes associated with AZS- and HFD-related obesity. When lambda.min = 0.0217, the LASSO regression model showed the best fit for AZS, selecting 6 core genes (AACS, C1QBP, MCRIP2, PPIL1, SERPINA3, ZGPAT), as shown in Fig. 2A. When lambda.min = 0.0088, the LASSO regression model showed the best fit for HFD obesity, with 4 core genes (C1QBP, MCRIP2, NAMPT, and PPIL1) selected, as shown in Fig. 2F.

Next, we constructed an SVM diagnostic model to estimate the predictive utility of the 9 shared key genes. The area under the ROC curve (AUC) for each model is shown in Fig. 2B-C. When the number of gene features was set to 7, the SVM model achieved the highest accuracy for AZS, identifying 7 optimal feature genes for AZS (C1QBP, AACS, ZGPAT, PPIL1, LOXL1, MCRIP2, and COL5A3). When the number of gene features was set to 3, the SVM model achieved the highest accuracy for HFD obesity, identifying 3 optimal feature genes for HFD obesity (NAMPT, PPIL1, C1QBP), as shown in Fig. 2G-H.

The overlapping genes identified by both machine learning methods were considered diagnostic biomarkers for both AZS and HFD obesity. We identified 2 diagnostic biomarkers: C1QBP and PPIL1. We subsequently evaluated the diagnostic performance of the core genes by plotting ROC curves. Internal dataset validation revealed that the core genes demonstrated significant diagnostic value for both diseases (AUC > 0.911). The GSE26982 and GSE8700 datasets were used as external validation sets for AZS and HFD obesity, respectively, and similarly showed excellent diagnostic value (AUC > 0.789), as shown in Figs. 2D-E and I-J.

### Core gene–transcription factor coexpression network

**Fig. 3:**
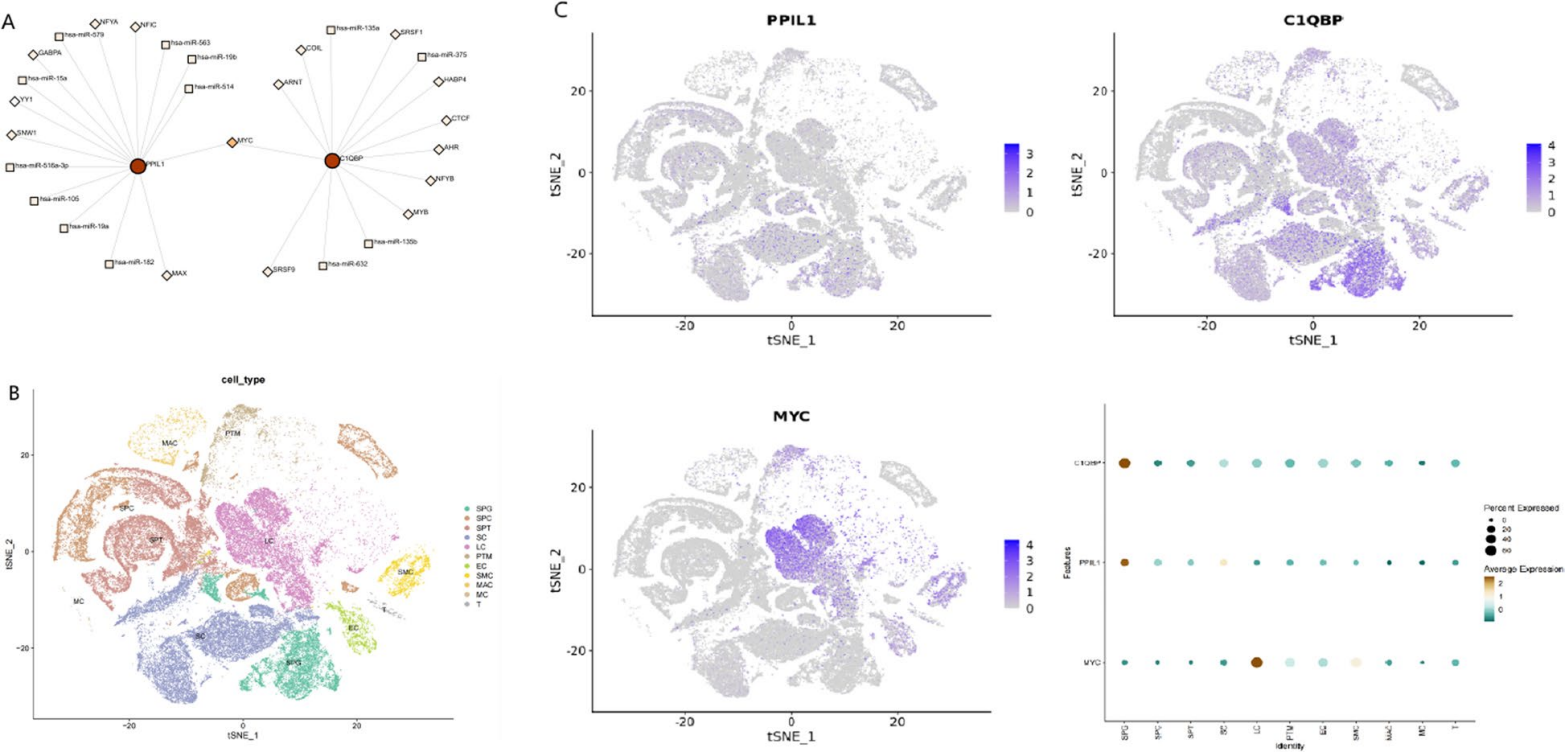
Core gene–transcription factor coexpression network (**A**) core gene–transcription factor coexpression network. **B** Testicular cell type profile of healthy males. **C** Expression and localization of the core gene–transcription factor coexpression network in testicular tissue cell populations. Note: SPG: spermatogonia; SPC: spermatocyte: SPT: spermatids/sperm; SC: Sertoli cell: LC: Leydig cell: PTM: parvubular myoid cell: EC: endothelial cells: SMC: wascular smooth muscle cell; MAC: macrophage; MC: mast cell; T: T cell

To investigate the regulatory mechanisms of the core genes, we used the igraph package in R to construct a core gene–transcription factor coexpression network, predicting the key upstream transcription factors that interact with the core genes. The results revealed that MYC serves as a bridge connecting the two core genes, potentially acting as the upstream transcription factor regulating both genes. We visualized the core gene–transcription factor coexpression network via Cytoscape software, as shown in Fig. 3A. To better understand the expression and localization of the core gene–transcription factor network within testicular tissue cell populations, we utilized the Male Health Atlas (MHA) database to explore the coexpression network features within testicular tissue, as depicted in Fig. 3B. Notably, C1QBP and PPIL1 were predominantly localized in spermatogonia, whereas MYC was primarily localized in stromal cells (Fig. 3C).

### GSEA analysis

**Fig. 4:**
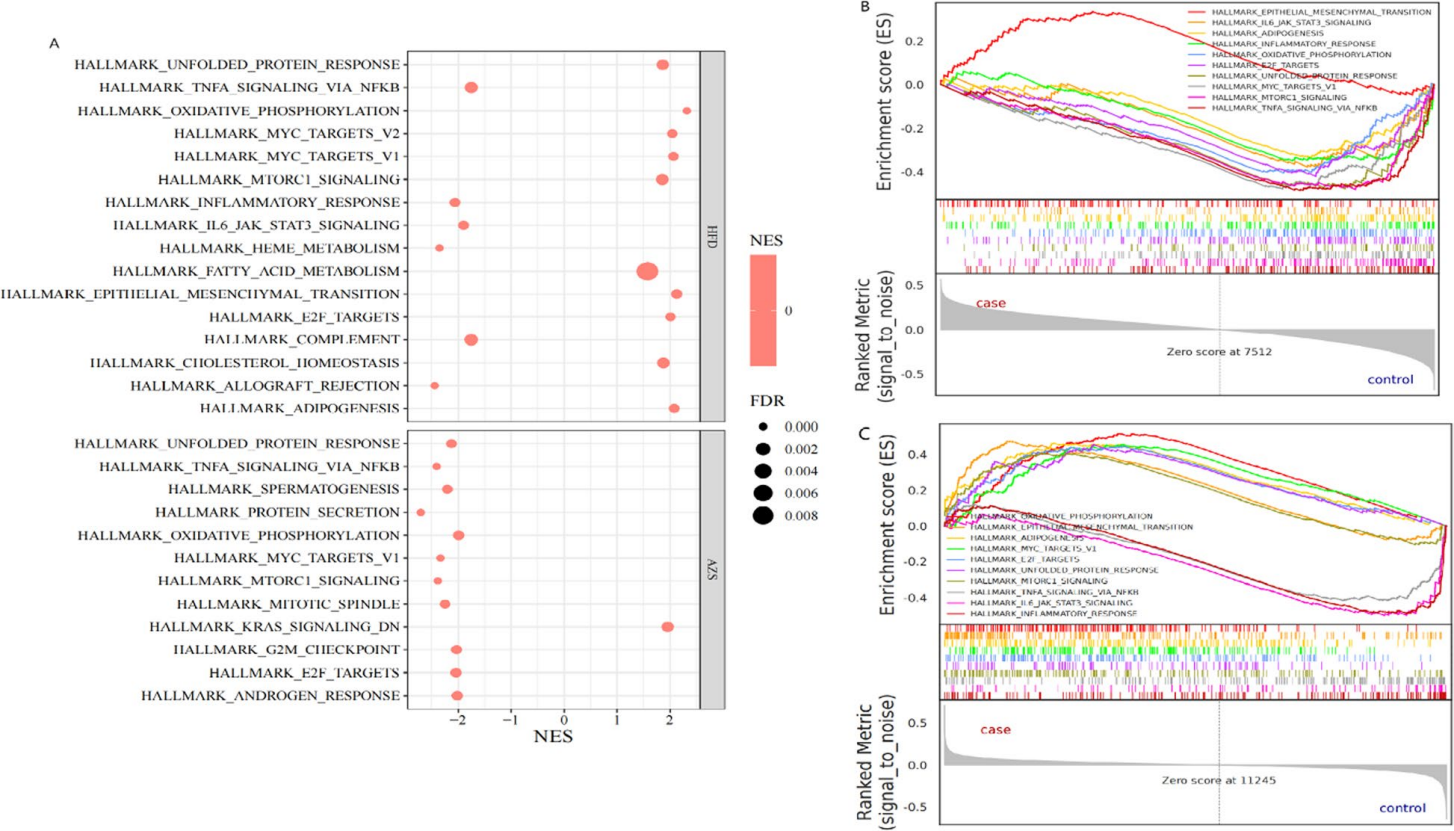
GSEA Analysis (**A**) GSEA results for HFD obesity and AZS (FDR < 0.01). **B**-**C** Shared GSEA entries for HFD obesity and AZS

Whole-genome GSEA revealed that both HFD-induced obesity and AZS were enriched in pathways related to cell growth, proliferation, and differentiation (the MTORC1 signalling pathway, MYC target signalling pathway, epithelial‒mesenchymal transition signalling pathway, and E2F target signalling pathway), energy metabolism (lipid biosynthesis and oxidative phosphorylation), cellular stress and homeostasis (unfolded protein response), and immune and inflammatory responses (the IL6 JAK-STAT3 signalling pathway, TNFα-NFKB signalling pathway, and inflammatory response). These findings suggest that these pathways may play a key role in both HFD obesity and AZS, as shown in Fig. 4.

### Experimental validation

**Fig. 5:**
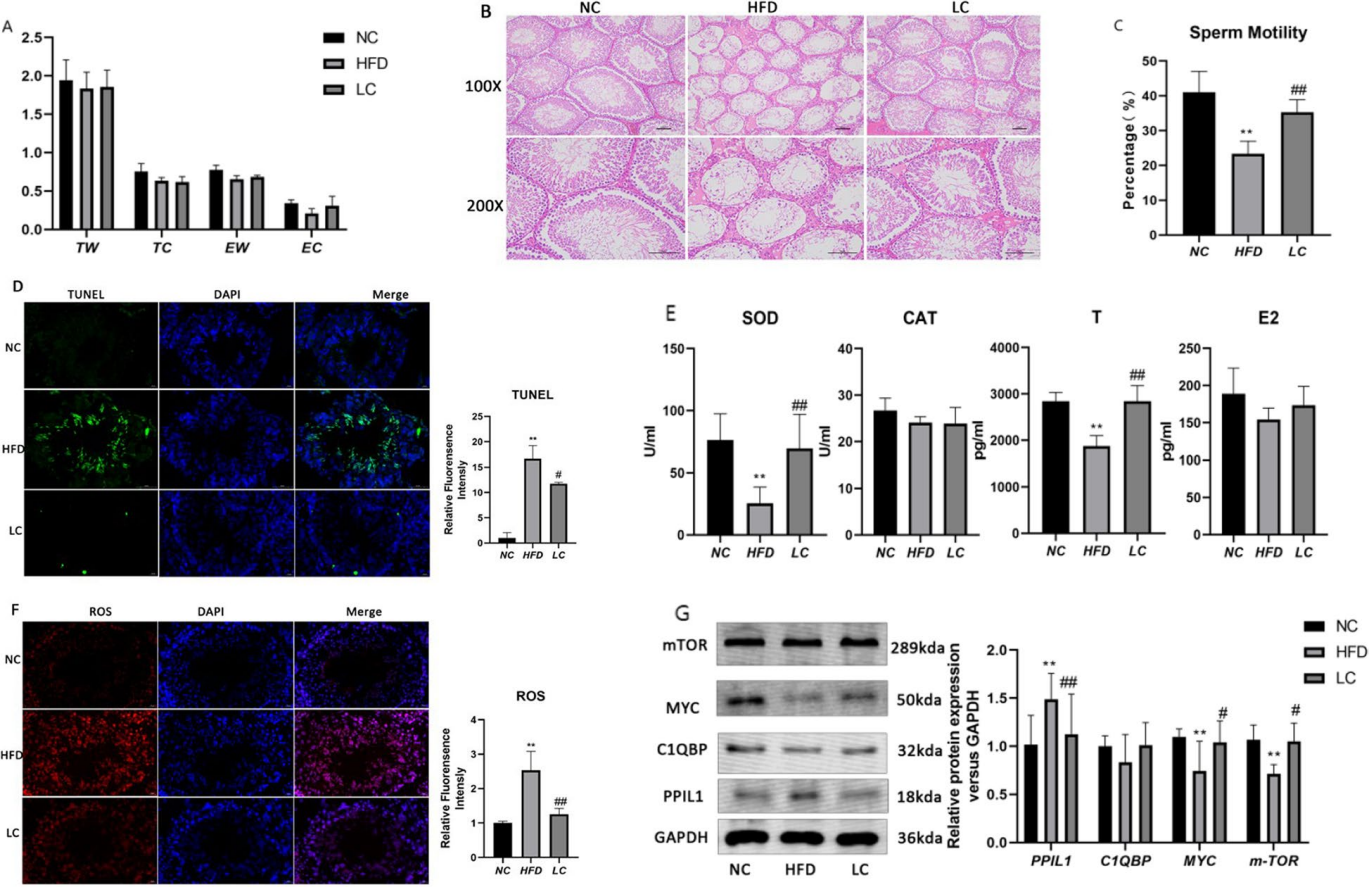
Experimental Validation Note: NC: Control group; HFD: high-fat diet obesity group; LC: L-carnitine group; SOD: superoxide dismutase; CAT: catalase; T: testosterone; E2: estradiol. *p < 0.05, **p < 0.01 compared with the NC group; #*p* < 0.05, ##*p* < 0.01 compared with the HFD model group. **A** Organ weight and index; **B** Testicular tissue H&E staining; **C** Sperm motility analysis; **D** Testicular tissue TUNEL; **E** Serum hormone and oxidative stress indicators by ELISA; **F** Testicular tissue ROS; **G** Testicular tissue Western blot analysis

This study validated the relationship between HFD obesity and AZS through animal experiments.Compared to the normal control (NC) group, the high-fat diet (HFD) group showed a decreasing trend in organ weights, though the differences were not statistically significant (HFD group: TW: 1.83 ± 0.22 vs NC group: 1.95 ± 0.26; EW: 0.66 ± 0.05 vs 0.78 ± 0.06; TC: 0.63 ± 0.04 vs 0.75 ± 0.11; EC: 0.21 ± 0.06 vs 0.34 ± 0.04; all indicators *P* > 0.05; Fig. 5A). Histological examination revealed significant structural lesions in the seminiferous tubules, characterized by disorganized arrangement and reduced spermatogenic cell density (Fig. 5B). High-fat diet-induced obesity significantly decreased sperm motility in rats (23.30 ± 3.68 vs 41.01 ± 6.01,*P* < 0.01; Fig. 5C) and significantly increased testicular apoptosis rate (16.70 ± 2.53 vs 1.00 ± 1.07, *P* < 0.01), suggesting that HFD-induced obesity mediates asthenozoospermia. Furthermore, the HFD group showed significantly reduced serum testosterone levels (1872.00 ± 230.10 vs 2842.00 ± 190.20,*P* < 0.01), significantly elevated reactive oxygen species (ROS) activity (2.54 ± 0.55 vs 1.00 ± 0.05,*P* < 0.01), and significantly decreased superoxide dismutase (SOD) activity (25.47 ± 13.06 vs 76.26 ± 21.12,*P* < 0.01), indicating a state of oxidative stress (Fig. 5D–F).

At the molecular level, Western blot results demonstrated that the HFD group had significantly downregulated MYC (0.75 ± 0.31 vs 1.10 ± 0.11, *P* < 0.01) and mTOR (0.71 ± 0.09 vs 1.07 ± 0.15, *P* < 0.01) protein expression, while PPIL1 expression was significantly upregulated (1.49 ± 0.27 vs 1.02 ± 0.30, *P* < 0.01). Although C1QBP expression showed a decreasing trend in the HFD group (0.83 ± 0.29 vs 1.00 ± 0.11), the difference was not statistically significant (*P* > 0.05; Fig. 5G).

L-carnitine (LC) intervention significantly reversed the adverse effects induced by HFD. Compared to the HFD group, LC treatment significantly improved sperm motility (35.26 ± 3.64% vs 23.30 ± 3.68%, *P* < 0.01) and reduced testicular apoptosis rate (11.73 ± 0.31% vs 16.70 ± 2.53%, *P* < 0.05). Meanwhile, LC administration significantly increased serum testosterone levels (2841.00 ± 337.40 vs 1872.00 ± 230.10,*P* < 0.01) and effectively alleviated oxidative stress, as evidenced by significantly reduced ROS levels (1.25 ± 0.17 vs 2.54 ± 0.55, *P* < 0.05) and increased SOD activity (69.57 ± 27.49 vs 25.47 ± 13.06, *P* < 0.05). At the molecular level, LC intervention significantly upregulated MYC (1.04 ± 0.23 vs 0.75 ± 0.31, *P* < 0.05) and mTOR (1.05 ± 0.19 vs 0.71 ± 0.09, *P* < 0.05) protein expression, and significantly downregulated the abnormally high expression of PPIL1 (1.13 ± 0.42 vs 1.49 ± 0.27,*P* < 0.01). C1QBP expression also showed a recovering trend in the LC group (1.01 ± 0.23 vs 0.83 ± 0.29), though the difference remained statistically non-significant (*P* > 0.05). These results indicate that L-carnitine effectively ameliorates HFD-induced asthenozoospermia by regulating the MYC/mTOR/PPIL1 signaling axis and mitigating oxidative damage.

## Discussion

In this study, we identified that HFD-induced obesity contributes to asthenozoospermia (AZS) through the dysregulation of the MYC–PPIL1 network, a pathway not previously established in this context. Our integrated bioinformatics and experimental approach revealed that MYC, a key transcriptional regulator, is suppressed in HFD-induced AZS and likely acts as an upstream regulator of PPIL1, a spliceosome component whose role in male infertility had been largely unexplored.

Beyond confirming the involvement of MYC, a more striking novel finding of our work is the identification of PPIL1 as a significantly upregulated gene in HFD-induced AZS. While PPIL1 is recognized for its role in pre-mRNA splicing and neurodevelopment [22], its involvement in obesity-related male infertility is, to our knowledge, reported here for the first time. The upregulation of PPIL1, coupled with the suppression of its potential upstream regulator MYC, suggests a previously unrecognized regulatory axis that may disrupt RNA processing and gene expression during spermatogenesis under obese conditions [23]. This finding expands the molecular landscape of AZS beyond the well-documented pathways of oxidative stress and hormonal imbalance.

Our results also reinforce the crucial role of MYC and mTOR signaling in maintaining sperm motility. The significant downregulation of both MYC and mTOR in our HFD model aligns with previous studies linking their activities to spermatogonial stem cell self-renewal and energy metabolism in sperm [10, 24–26]. However, our study adds a new layer to this understanding by positioning MYC not only as a downstream effector of metabolic stress but also as a potential master regulator connecting HFD-induced obesity to specific molecular effectors like PPIL1. The co-expression network analysis strongly supports this hierarchical relationship, providing a more precise mechanistic hypothesis for future investigations.

The role of C1QBP in our model was less clear. Although it was identified as a core gene bioinformatically and is known to be involved in mitochondrial function and lipid homeostasis [27–29], its protein expression changes were not statistically significant in our animal validation. This discrepancy suggests that C1QBP's role might be context-dependent or regulated at a post-transcriptional level, warranting further study to clarify its contribution to the phenotype.

Importantly, we demonstrated that the antioxidant L-carnitine could partially reverse the HFD-induced impairments, not only restoring sperm motility and mitigating oxidative stress but also normalizing the expression of the key molecules in the MYC–PPIL1 axis. This therapeutic effect provides functional evidence for the pathophysiological relevance of this network. The ability of L-carnitine to modulate MYC and PPIL1 expression highlights the plasticity of this pathway and its potential as a target for intervention [13–15, 30].

In conclusion, our findings propose a novel mechanism for HFD-induced AZS, centered on the MYC–PPIL1 regulatory network. This study moves beyond the established paradigms of general oxidative stress and hormone imbalance to pinpoint a specific transcriptional-splicing axis that is disrupted in obesity-related male infertility. The partial rescue by L-carnitine underscores the therapeutic potential of targeting this pathway. Future research should focus on elucidating the precise molecular interactions between MYC and PPIL1 and validating this axis in human studies.

## Conclusion

This study not only confirmed the close relationship between HFD-induced obesity and AZS but also revealed its potential molecular mechanisms, particularly the regulatory role of the MYC‒PPIL1 network. These findings provide new molecular evidence for further exploration of infertility caused by obesity and offer novel insights and potential strategies for future interventions and treatments, such as the application of antioxidants.

Despite these novel findings, our study has several limitations. First, the animal experiment was conducted with a relatively small sample size, which, while sufficient to detect robust phenotypic and molecular changes, warrants caution in interpretation and necessitates validation in larger-scale studies. Second, the precise molecular interaction between MYC and the PPIL1 promoter requires further validation through techniques such as chromatin immunoprecipitation (ChIP) assays. Third, the functional role of PPIL1 in spermatogenesis remains to be fully elucidated.

In summary, our work establishes the MYC–PPIL1 network as a crucial link between HFD-induced obesity and AZS. Future research should focus on replicating these findings with increased statistical power, delineating the detailed mechanisms of this axis, and exploring its potential as a diagnostic biomarker and therapeutic target for obesity-related male infertility.

## Data Availability

The data are available from the corresponding author upon reasonable request.
